# Is iodized salt efficient to overcome iodine deficiency in pregnants?

**DOI:** 10.4274/tjod.galenos.2020.20727

**Published:** 2020-07-29

**Authors:** Nazlı Nur Aslan Çin, Neslihan Bezirganoğlu Altuntaş, Ayşe Özfer Özçelik

**Affiliations:** 1 Ankara University Faculty of Health Sciences, Department of Nutrition and Dietetics, Ankara, Turkey; 2 University of Health Sciences Turkey, Trabzon Kanuni Training and Research Hospital, Clinic of Gynecology and Obstetrics, Trabzon, Turkey

**Keywords:** Iodine deficiency, iodine intake, urinary iodine concentration, pregnant women

## Abstract

**Objective:**

Iodine is a trace element that synthesizes thyroid hormones necessary for optimal human growth and development. The relationship between dietary iodine intake and spot urinary iodine excretion in pregnant women has not been previously evaluated in Trabzon city, which is an endemic area of iodine deficiency in the Black Sea region of Turkey. This study aimed to evaluate the relationship between dietary iodine intake and urine iodine excretion in pregnant women.

**Materials and Methods:**

This study enrolled 150 pregnant women aged between 19 and 45 years who applied to Clinic of Gynecology and Obstetrics in Trabzon. Spot urine specimens were taken, and dietary iodine intake data were collected using a food frequency questionnaire (FFQ) and 24-hours dietary recall (24-h DR) method.

**Results:**

The median urinary iodine concentration (UIC) in the general specimen was 100.6 μg/L. Of the pregnant women, 80.0% had insufficient and 20.0% had sufficient iodine levels, according to UIC. Although total iodine-rich food intake determined by FFQ was sufficient in 20.7% (n=31) of participants, 24-h DR iodine intake was sufficient only 10.7% (n=16). A significant association between urinary iodine excretion and iodine intake was observed in both 24-h DR and FFQ intake estimates (p<0.05). The iodine intake values obtained in both 24-h DR and FFQ and the iodized salt effect were correlated with UIC in all models (p<0.05). Even though 96.0% of pregnant women used iodized salt, its effect on UIC was 15.2%.

**Conclusion:**

Both methods indicate that the iodine intake of pregnant women might be insufficient in Trabzon area. Also, although iodized salt use is high in pregnant women in Trabzon, it is not enough to prevent iodine deficiency.

**PRECIS:** The use of iodized salt is high in pregnant women, but consuming iodized salt alone could not prevent iodine deficiency.

## Introduction

Iodine, which is a trace element of the human body (15-20 mg), ensures the optimal growth and development of a newborn. The sufficiency of thyroid hormones [thyroxine (T4) and triiodothyronine (T3)], particularly during pregnancy, is vital for the cerebral and neurological development of the fetus^(1)^. Therefore, iodine-rich dietary is crucial during pregnancy, and milk and fish are the leading supplies of iodine in meals. Besides, iodized salt is a strategy developed in many societies to control and prevent iodine deficiency^(2)^.

Measuring urinary iodine concentration (UIC) is recommended to assess the dietary iodine intake during pregnancy. Concerning the urinary excretion of iodine in pregnant women, the World Health Organization (WHO) defines 150-249 µg/L as “sufficient” and <150 µg/L as “insufficient^(3)^.” The renal clearance of iodine increases around 30-50% during pregnancy, which causes iodine deficiency, leading to an increase in the daily iodine need of pregnant women compared with normal adults. Moreover, the increased renal clearance of iodine and concurrent and continuous increase in the production of thyroid hormone stimulate the thyroid, as well. The increased level of stimulation during pregnancy is more common in areas suffering from iodine deficiency, which leads to abortion and stillbirth^(1)^.

Turkey has been listed among the countries with sufficient amount of iodine, due to the addition of iodine to salt in recent years^(4)^. However, a research performed in 2011-2012 in Trabzon, a city reported having sufficient iodine intake level, assessed UIC in a large group of pregnant women (n=864) and found 77.9% iodine deficiency^(5)^. Most of the pregnant women who live in areas with iodine deficiency or with low iodine intake can attain the sufficient level by either taking iodine supplements or consuming iodine-rich food^(6)^. However, in the endemic region of Trabzon, dietary iodine intake of pregnant women has not been evaluated so far. This study distinguishes from previous reports by investigating iodine intake with both food frequency questionnaire (FFQ) and 24-hours (24-h) recalls. Therefore, this study aimed to assess the dietary iodine intake and urinary iodine excretion of pregnant women aged between 19 and 45 years in Trabzon, where there is an endemic area of iodine deficiency, and to detect the reliability of both methods by comparing UIC.

## Materials and Methods

### Study Design

This research is a descriptive, cross-sectional study. The number of participants recruited was calculated with reference to the study by Alvarez-Pedrerol et al.^(7)^ According to the results of different power analyses among two independent groups (milk intake frequency and UIC), a minimum of 130 individuals were necessary to achieve a type 1 error (a)= 0.05, effect size of 0.5, and power of 80%. The study recruited 150 pregnant women between 19 and 45 years old. A central state hospital was chosen as the research place; hence, we could assess individuals from various socioeconomic degrees. Approval of the Ethics Committee was received from Ankara University Faculty of Medicine (approval number: 11-478-16, date: 23.06.2016), and the Helsinki Declaration principles were followed in the research. Before the application of the survey, each participant was informed about the contents of the research, and they were asked to sign the informed consent forms, indicating that they voluntarily agreed to participate in the research.

The research participants were selected among pregnant women aged between 19 and 45 years, with no thyroid disease history or thyroid treatment medications. Research data were collected using a questionnaire form and face-to face interviews. The intelligibility of the questions was tested with 10 women, and the survey form was finalized after the necessary corrections were made. The survey form included general information about the pregnant women (e.g., age, education level, time passed since previous delivery, and prenatal nutritional support), their iodine level intake through food, and their iodized salt consumption behaviors. In addition, spot urine specimens were taken from the women to assess the urinary iodine level.

### Dietary Iodine Intake

The FFQ, which consisted of 50 semi-quantative items, was used to assess only iodine-rich and possible goitrogenic food consumption in the last 1 month. The questionnaire is an adapted version of the FFQ developed by Willett et al.^(8)^, and it has already been used and validated in the general population in Turkey^(9)^. The frequency of food and iodine salt intake was evaluated per day, week, or month. The “A Photographic Atlas of Food Portion Sizes” developed for Turkey was used to correctly assess the amount of food consumed^(10)^. Data were collected regarding women’s consumption of seafood (processed fish, lean fish, fatty fish, and shellfish), meat and poultry (processed meat, red meat, and poultry), possible goitrogenic food (cruciferous vegetables; cabbage, kale, kohlrabi, cauliflower, spinach, radish, broccoli, brussel sprouts, turnips, and sweet potatoes), egg, milk, and other dairy products (cheese, yogurt, and butter). Responses were divided into weekly categories for seafood, meat, poultry, and goitrogenic foods and daily for milk, yogurt/ayran, cheese, and egg. Milk, yogurt/ayran, meat, and poultry intakes were divided into quartiles. Other food intake was taken according to the recommended portion in the Turkey Dietary Guideline^(11)^.

Iodine salt intake was reported based on standard referent portion sizes as pieces and spoons. The WHO^(3)^ and TUBER^(11)^ recommend less than 6 g (approximately 2400 mg) of iodized salt consumption per day for adults. In this study, 6 g/day was considered the cut-off value for salt intake.

Total iodine-rich food intake with FFQ and 24-h dietary recall (24-h DR) was calculated using iodine content in the food indicated in the United States Department of Agriculture (USDA) food composition database^(12)^. Only some types of fish, shellfish, and iodized salt were calculated from the Turkish food composition database^(13)^. According to the WHO, the dietary intake of iodine in pregnant women was evaluated as “≤250 µg/100 g insufficient” and “≥250 µg/100 g sufficient^(3)^.”

### Laboratory Analysis

Spot urine specimens were collected from the pregnant women who agreed to participate in the research. Urine specimens were first put into medium flow urine deionized plastic containers and then into two tubes of 2 mL deiodized capped tubes. The specimens were delivered to the biochemistry laboratory of the hospital on the same day, and all urine specimens were stored at -20 ºC for 2 weeks in a deep freezer until the day of analysis. Urine samples were centrifuged for 3 min at 1500 rpm in a Hettich Micro 200R centrifuge before the study. The resulting supernatants were collected, and the urine iodine level was measured in the biochemistry laboratory by calorimetric method, which depends on the Sandell-Kolthoff reaction using the arsenic acid solution. Urine iodine levels were given in µg/L.

Urine iodine levels of the pregnant women were assessed as determined by the WHO, and the assumptions were accepted as follows: “<150 µg/L insufficient” and “≥150 µg/L sufficient.”

### Statistical Analysis

Statistical Package for the Social Sciences 22.0 package program was used to evaluate the data. UIC, dietary intake of most nutrients, and food groups were not normally distributed. The median and 95% reliability range values are presented. Mann-Whitney U test was used to assess the UICs and total iodine-rich food intake between the two groups that did not display a normal distribution, and Kruskal-Wallis variance analysis to evaluate the UICs and total iodine-rich food intake among the three groups. Pearson’s correlation coefficients were used to examine the associations between UIC and total-iodine rich food intake with FFQ and 24-h DR. Factors that may be associated with UIC (age groups, trimester, iodized salt intake, total iodine-rich food intake, and 24-h DR iodine intake) were evaluated with linear regression analysis. In all statistical tests, the range of reliability was accepted as 95.0% and evaluated at significance level of p*<*0.05.

## Results

The sociodemographic characteristics of pregnant women according to UIC and their total iodine-rich food intake are shown in Table 1. The median UIC in the general specimen was 100.6 (range: 22.7-483.0) µg/L. The mean age of the pregnant women was 28.6 (between ages 19-42) years, and the UIC and iodine-rich food intake of the pregnant women at 19-25 years of age was lower than the other age groups, but there was no significant difference (p>0.05). Both UIC and iodine-rich food intake were significantly higher in the first and second trimesters than in the third trimester (p<0.05). However, the level of education, number of pregnancies, and region of residence did not significantly differ between UIC and iodine-rich food intake (p>0.05).

UIC levels with food consumption of the pregnant women are provided in Table 2. While 60.7% of the pregnant women participating in the research reported that they never drank milk, the women consuming more than 190 mL of milk per day had a median UIC of 93.7 µg/L, which indicates that there is no statistically significant difference (p>0.05*). *While the women consuming more than one serving of white cheese daily had a high median UIC compared with those consuming less than one serving, the difference was not statistically significant (p>0.05). The difference between meat, poultry, and fish consumption and the UIC was found to be statistically insignificant. Similarly, daily yogurt/ayran and egg intakes were not significant with UIC (p>0.05). Of the pregnant women, 96.0% were consuming iodized salt, and those who consumed more than 6 g of iodized salt every day were found to have a significantly higher median UIC than those who did not (p<0.01). It was determined that the participants with sufficient iodine-rich food intake were significantly higher than those with insufficient UIC levels (p<0.01).

**Figure 1:**
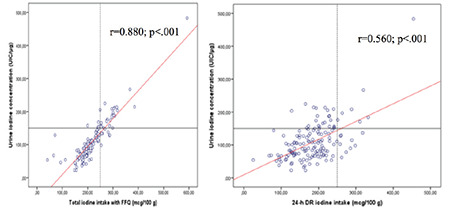
Correlation between urinary iodine concentration (µg/mL), total iodine intake with food frequency questionnaire, and 24-hours dietary recall iodine intake (mcg/100 g)

The correlation between UIC and total iodine-rich food intake of pregnant women are shown in Figure 1. According to the WHO criteria, 80.0% of the pregnant women had insufficient and 20.0% had sufficient iodine levels according to UIC (1.3% of them had high UICs of ≥250 µg/L). Although total iodine-rich food intake determined by FFQ was sufficient in 20.7% (n=31) of participants, 24-h DR iodine intake was sufficient only in 10.7% (n=16). There was a strong positive correlation between FFQ total iodine-rich food intake and UIC (r=0.880, p<0.001). Similarly, 24-h DR iodine intake had a significant positive correlation with the UIC (r=0.560, p<0.001).

When factors that could affect UIC (age group, trimester, iodized salt intake, FFQ iodine intake, and 24-h DR iodine intake) were evaluated with linear regression analysis, all models were deemed important for UIC (R^2^= 0.872, 0.872, and 0.871, respectively, p<0.05). It was determined that age groups and trimester were not related to UIC in Model I (p>0.05), but total iodine-rich food determined by FFQ, iodized salt, and 24-h DR iodine intake affected all models for the UIC (p<0.05). When Model III was analyzed, it was found that every 1-unit increase in iodized salt consumption affects UIC by 15.2%, whereas the FFQ iodine intake affects by 77.0%. Unlike FFQ iodine intake, the effect of iodine intake on UIC was less (7.1%) with a 24-h DR (Table 3).

## Discussion

UIC is deemed to be a good indicator, reflecting the recent level of iodine in pregnant women^(14)^. We found that pregnant women living in Trabzon province of the Black Sea region of Turkey may have insufficient amount of iodine, according to the UIC criteria. Furthermore, a significant association between urinary iodine excretion and iodine intake was observed in both 24-h DR and FFQ intake estimates. In this research, as reported in previous studies held in Turkey, it was determined that pregnant women failed to meet their increasing iodine need. This may have a potential negative effect on fetal brain development^(5)^.

In studies conducted with small samples taken from pregnant women living in different areas of Turkey, the UIC level varied from 77.4 to 149.7 µg/L^(15,16,17,18)^. In studies conducted on iodine deficiency (80% iodine deficiency), the level was found to change between 49.6% and 100%^(5,15,16,17,18)^, similar to this research. Previous studies indicate that in areas where iodine intake has become sufficient, iodine deficiency was still a serious problem for pregnant women.

Although Trabzon city center had previously been an endemic area, it was sufficient in terms of median UIC in school-aged children. However, UIC levels of pregnant woman in their first and second trimesters were higher than in the third trimester. Similarly, studies conducted with larger specimens also supports these results^(5,15)^. However, there are some other studies reporting that UIC increased as gestational weeks have passed^(17,18)^. As the results of this research showed lower median UIC levels in pregnant women as opposed to other studies, it is considered that as the gestational weeks pass, the amount of iodine to be transferred to the fetus will increase as well, decreasing the urinary iodine excretion. Besides, decreased iodine intake of pregnant women may lead to insufficiency as the trimester increases.

The pregnancy period is a unique process that can be affected by several factors. In this research, it was found that the level of median UIC increased as the age of the mother increased (p>0.05). This may occur due to two reasons. First, older women may consume more iodine-rich foods as in this study, or urinary creatine excretion might be decreasing as one ages, which is not evaluated in this research. Here, dietary iodine intake was shown increase with age (89.7, 205.9, and 213.5 µg/L for ages 19-25, 26-31, and ≥32 years; p>0.05). According to the findings of the Adult National Diet and Nutrition Survey, dietary iodine intake increases with age, and elder women have significantly higher iodine intake than young women^(19)^.

The use of iodized salt is reported to be the easiest, cheapest, and most effective method in the prevention of iodine deficiency in a society. According to the findings of the Demographic and Health Survey in Turkey^(20)^, although the use of iodized salt in a household was 70.2% in 2003, it increased to 85.3% in the 2008 Demographic and Health Survey^(21)^. In this research, 96.0% of pregnant women reported that they consume iodized salt, and the median urinary iodine excretion of pregnant women consuming iodized salt more than 6 g was significantly higher (p<0.05). Therefore, sole consumption of iodized salt seems to be insufficient (79.3%) to fulfill the increased iodine need in pregnancy. A research conducted in Albania reported that eventhough 99.6% of pregnant women (n=365) had consumed iodized salt for 11 years in a prophylaxis program, the median UIC was still 85 µg/L^(22)^. The quality and level of iodine became more important following the extensive options of iodized salt in the market. Recently, the amount of iodine labeled on the package of salts was reported to be different from the actual amount of iodine available. This difference may arise due to iodine losses, illegal production or lack of quality control, and bad packaging or post-packaging distribution problems^(23)^. Furthermore, consuming more than 6 g of iodized salt to achieve the sufficient level of UIC may increase the risk of certain diseases (pre-eclampsia, edema, hypertension, etc) in pregnant women.

Although iodized salt consumption has increased in Turkey recently, iodine deficiency is still a problem for pregnant women even in metropolitan cities such as Ankara and İstanbul^(17,18)^.

Relevant studies conducted in Europe and New Zealand have shown that women with iodine supplement intake had significantly higher UIC^(24)^. Here, all the participating pregnant women reported that they were not receiving iodine supplements. Iodine deficiency was detected despite the high rate of iodized salt consumption. Thus, reviewing the findings of other studies, adding a 150 µg elementary iodine containing a multivitamin tablet to the diet, in addition to iodized salt use, can be advised for pregnant women living in Turkey^(25,26)^.

Consumption of food that are low in iodine leads to iodine deficiency in pregnancy^(27)^. In a study conducted in Italy, cow’s milk was found to be a good source of iodine, and iodine supplements were recommended for those who did not consume milk^(28)^. Another study in the UK assessed the 24-h iodine excretion of those consuming more milk, eggs, and seafood and found a significantly higher iodine excretion. However, milk and iodine supplement intake in pregnant women is reported to be a more important indicator of iodine levels in pregnant women^(2)^. This study found a higher median UIC of pregnant women consuming more than 190 mL milk per day (p>0.05). As milk containers are not contaminated with iodophors in our country, unlike in other studies, it was thought that there was no relationship between milk consumption and urinary iodine excretion.

In our study, we observed restricted effect of fish intake on UIC. Similarly, it was reported that fish/seafood intake had no significant influence on 24-h urinary iodine excretion in Norway^(29)^. Iodine content of fish and seafood is known to be high, but their contribution to the overall dietary iodine intake is mild unless consumed every day^(1,30)^.

Excessive intake of goitrogens in food prevents iodine from bonding with the thyroid hormone precursor, tyrosine, and suppresses T4 secretion^(1)^. Bath et al.^(2)^ reported no significant relationship between goitrogenic food consumption and UIC. Also in this research, no significant difference was found between goitrogenic food and UIC levels. The amount of iodine in food may change depending on the season and the iodine level in soil. The research was conducted in summer, which was not the season for growing goitrogenic food such as black cabbage, radish, and turnips, which might have affected the UIC.

As more than 90% of dietary iodine is excreted in the urine by the kidneys, the most appropriate indicator reflecting iodine intake is urinary iodine excretion^(1)^. Urinary iodine excretion varies within the day and peaks after the main meals. In this study, UIC was more closely correlated with FFQ (total iodine-rich food) than with 24-h DR iodine intake estimates. Nevertheless, Brantsaeter et al.^(29) ^reported a higher corelation between UIC and 24-h DR than FFQ iodine intake estimates.

### Study Limitations

First, this was a cross-sectional study, so the study design may biased the results.

Furthermore, the small sample size may not reflect the situation in all pregnant women in the region. We are aware that there are limitations of using single-spot urine specimens. UIC alters throughout a day so that would be a more precise indicator to collect a 24-h urine specimen or multiple samples in different hours of a day^(30)^. However, 24-h urine specimens are inconvenient for the participants and difficult to collect accurately, and that was not possible to obtain several urine specimens. The amount of iodine in food is affected by various factors (geography and climatic conditions). Therefore, collecting the data of the study only in summer months might have caused an alteration in the results. USDA data have been used because of the unknown iodine content in food (excluding some fish types, shellfish, and iodine salt) in Turkey. The iodine content of food grown in each country is different^(1)^. Future studies are suggested to define iodine content of food in Turkey. To our knowledge, this study separates from the previous ones as being the first to compare UIC with two dietary iodine intake record methods (FFQ and 24-h DR).

## Conclusion

In Trabzon, iodine deficiency was observed in pregnant women. Although other studies determined milk and iodine supplement intake as the best indicators to assess the iodine level in pregnant women, milk had no significant effect on the iodine level in this study. As recommended by the current world health policies, consuming two to three servings of milk per day and one to two servings of fish per week would provide a sufficient amount of iodine support. In Turkey, the amount of iodine in food can be increased by adding iodine to such food. Moreover, consuming iodized salt alone could not prevent iodine deficiency. Therefore, addition of iodine to vitamin-mineral supplements during the prenatal and pregnancy periods may improve iodine levels. Iodine deficiency in pregnant women still seems to be a major public health problem in certain cities of Turkey, and there is no comprehensive epidemiological study comparing dietary iodine intake and urinary iodine excretion in pregnant women.

## Figures and Tables

**Table 1:**
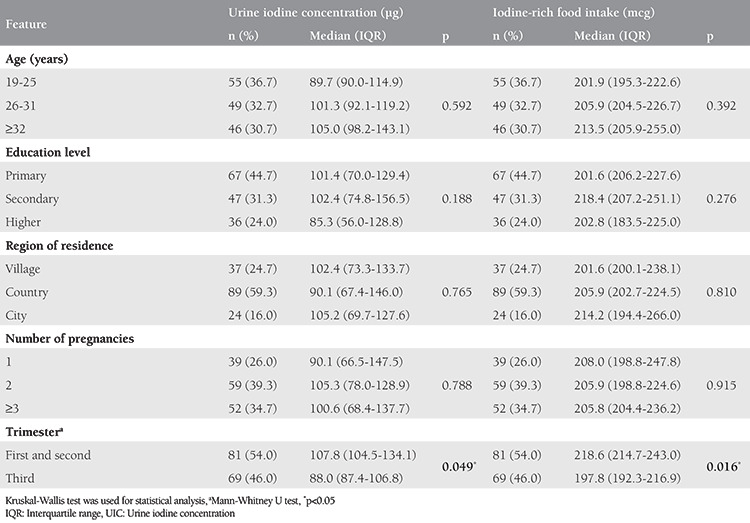
Urine iodine concentration (μg) and total iodine-rich food intake (mcg) with food frequency questionnaire according to sociodemographic characteristics

**Table 2:**
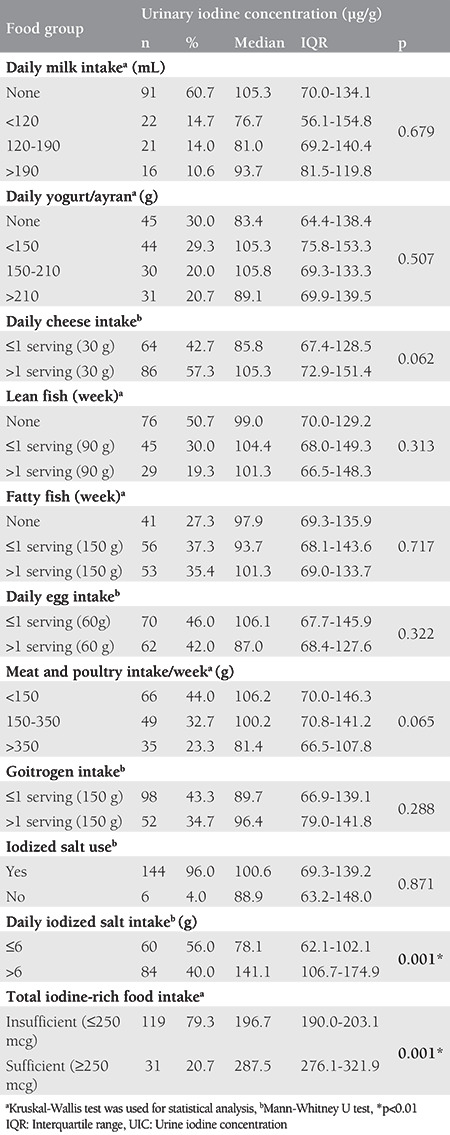
Urine iodine concentration (μg) according to food consumption

**Table 3:**
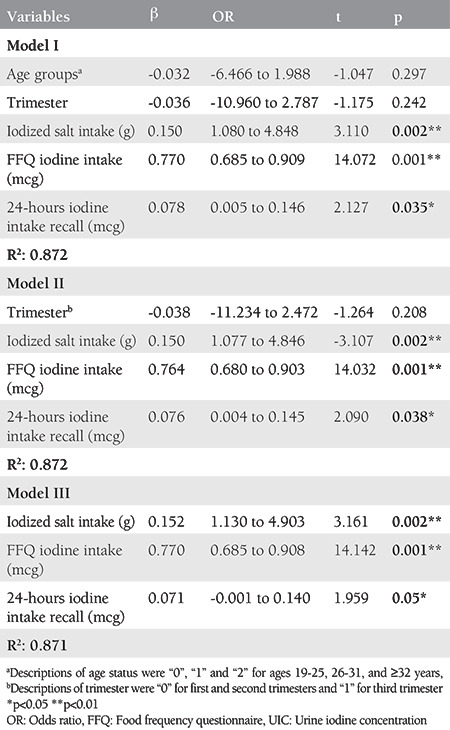
Urine iodine concentration (μg) linear regression analysis
